# Novel hematologic ratios and systemic inflammation index in ADHD: effects of methylphenidate treatment

**DOI:** 10.3389/fpsyt.2025.1621767

**Published:** 2025-07-16

**Authors:** Meryem Kaşak, Hande Günal Okumuş, Yusuf Selman Çelik, Fatma Zehra Kırşan, Yusuf Öztürk, Ayşegül Efe

**Affiliations:** ^1^ Department of Child and Adolescent Psychiatry, Ankara Etlik City Hospital, Ankara, Türkiye; ^2^ Department of Child and Adolescent Psychiatry, Uşak Training and Research Hospital, Uşak, Türkiye; ^3^ Department of Child and Adolescent Psychiatry, Dörtyol State Hospital, Dörtyol, Hatay, Türkiye

**Keywords:** attention deficit hyperactivity disorder, inflammation, methylphenidate, blood parameters, NHR, SII

## Abstract

**Background:**

Attention-deficit/hyperactivity disorder (ADHD) is a common neurodevelopmental disorder, and recent research suggests systemic inflammation contributes to its pathophysiology. This study aimed to evaluate novel inflammatory markers—neutrophil-to-HDL ratio (NHR), lymphocyte-to-HDL ratio (LHR), monocyte-to-HDL ratio (MHR), platelet-to-HDL ratio (PHR), and systemic immune-inflammation index (SII)—in children with ADHD compared to healthy controls. Additionally, it assessed changes in these markers after 12 weeks of long-acting methylphenidate treatment and potential differences among ADHD subtypes.

**Methods:**

This prospective study included 114 newly diagnosed, treatment-naive ADHD patients (aged 6–12) and 52 matched controls. Blood samples were obtained at baseline and after 12 weeks of treatment. Inflammatory markers were calculated from complete blood count and HDL levels. ADHD symptom severity was assessed using the Conners Parent Rating Scale-Revised: Short Form (CPRS-R:S), and anxiety and depression were measured with the Revised Child Anxiety and Depression Scale (RCADS).

**Results:**

ADHD patients showed significantly elevated baseline levels of NHR, LHR, MHR, PHR, and SII compared to controls (Cohen’s d range = 0.17–0.69). NHR independently predicted ADHD. Post-treatment, all inflammatory markers significantly decreased, suggesting a potential anti-inflammatory effect of methylphenidate (Cohen’s d range = 0.17–0.91). Post-treatment LHR was higher in the combined ADHD subtype.

**Conclusions:**

This study underscores inflammation’s role in ADHD and suggests these markers may reflect systemic inflammation in ADHD, but their clinical utility requires further investigation.

## Introduction

Attention-deficit/hyperactivity disorder (ADHD) is a prevalent neurodevelopmental condition affecting approximately 5.3% of the pediatric population. It is characterized by age-inappropriate inattention, hyperactivity, and impulsivity levels that significantly impair daily functioning (1). The etiology of ADHD remains unclear, but it is believed to result from complex interactions between genetic and environmental factors. Genetic influences are substantial, with heritability estimates ranging from 70% to 80% (2). In addition, emerging evidence highlights the role of stress-related systems, particularly neuroinflammation, in the pathophysiology of ADHD (3).

Neuroinflammation is hypothesized to contribute to the etiology of ADHD by disrupting neurodevelopmental processes through early-life alterations in microglia, astrocytes, cytokines, chemokines, oxidative stress, and associated molecular pathways (4). Peripheral pro-inflammatory cytokines, driven by heightened inflammatory responses and an imbalance between pro-inflammatory and anti-inflammatory mechanisms, can access the brain via humoral and neural pathways. Once in the brain, these cytokines perpetuate inflammatory cascades through neuroimmune interactions, further influencing neurodevelopmental trajectories. (5). Altered cytokine levels have been reported in both cerebrospinal fluid and peripheral blood samples of children with ADHD. For instance, studies have demonstrated significantly elevated serum IL-6 levels and reduced TNF-α levels in children with ADHD, indicating pro-inflammatory activity (6). Furthermore, multiple studies have suggested a relationship between inflammatory markers and ADHD severity, with elevated inflammatory markers often associated with more pronounced symptoms (7). However, conflicting evidence exists, as some research has reported no significant differences in inflammatory biomarkers in patients with ADHD (8).

Despite the significant emphasis on inflammation in ADHD research, most studies have primarily focused on biomarkers such as cytokines, chemokines, and oxidative stress markers. Nevertheless, the clinical utility of these biomarkers remains constrained by their limited availability, high costs, and the complexity of their measurement procedures (9). As an alternative, inflammatory markers derived from routine complete blood count (CBC) parameters, such as leukocyte and platelet levels, offer a cost-effective and widely accessible option. These parameters also reflect the dynamic immune response central to inflammation (10). High-density lipoprotein (HDL), which exerts antioxidant and anti-inflammatory effects by reducing cytokine release from monocytes and macrophages, is another promising biomarker (11). Given the roles of leukocytes, platelets, and HDL in inflammation, ratios such as the neutrophil-to-HDL ratio (NHR), lymphocyte-to-HDL ratio (LHR), monocyte-to-HDL ratio (MHR), and platelet-to-HDL ratio (PHR) have recently emerged as novel markers for systemic inflammation and oxidative stress. These ratios have demonstrated potential utility in various diseases (9, 12, 13). However, while studies examining these novel inflammation ratios in psychiatric disorders such as bipolar disorder (14–16), schizophrenia (9, 17) and depressive disorder (18, 19) are increasing, no such research has been conducted in ADHD. NHR, LHR, MHR, and PHR may serve as more reliable biomarkers by integrating the anti-inflammatory and antioxidant properties of HDL with the pro-inflammatory effects of leukocyte and platelet levels, making them valuable indicators of inflammation and oxidative stress (16).

Interestingly, the systemic immune-inflammation index (SII), a ratio initially developed for tumor assessment (20), has emerged as a promising tool for quantifying immune response and inflammation (21). SII combines three peripheral inflammatory cell counts—neutrophils, lymphocytes, and platelets—and has demonstrated utility in detecting inflammation in psychiatric disorders (9, 22, 23). Notably, a recent study reported a positive correlation between SII and the severity of hyperactivity symptoms in adults with ADHD (22).

Methylphenidate (MPH) is the first-line pharmacological treatment for ADHD in children and adolescents (24). MPH exerts its effects primarily by targeting dopamine and norepinephrine transporters (DAT and NET). It also interacts with adrenergic and AMPA receptors and modulates serotonin, glutamate, and GABA signaling (25). While the mechanisms underlying MPH’s therapeutic effects are complex and not fully understood, its ability to alleviate core ADHD symptoms is well established. However, studies examining the impact of MPH on oxidative stress, apoptosis, and inflammation markers—factors implicated in the etiology of ADHD—have yielded inconsistent results (3, 26). Therefore, the relationship between MPH treatment and inflammatory mechanisms in human plasma warrants further investigation.

This study hypothesizes that children with ADHD exhibit a pro-inflammatory state compared to healthy individuals and that MPH treatment is associated with an anti-inflammatory profile. Therefore, the primary aim was to evaluate differences in NHR, MHR, LHR, PHR, and SII levels between untreated children with ADHD and healthy controls at baseline. Furthermore, we sought to assess changes in NHR, MHR, LHR, PHR, and SII levels before and after 12 weeks of treatment with long-acting MPH in children with ADHD. Lastly, we aimed to explore whether these inflammatory markers differed between ADHD subtypes before and after treatment.

## Materials and methods

### Participants

This study was conducted in 2024 at the Child and Adolescent Psychiatry outpatient clinic of Ankara Etlik City Hospital. A total of 166 children (113 boys and 53 girls), aged between 6 and 12 years (mean age: 8.89 ± 1.79 years), were included in the study. The study employed a prospective and open-label design.

ADHD patients and healthy control groups were determined based on inclusion and exclusion criteria. Inclusion criteria for the patient group were as follows: (a) age between 6 and 12 years, (b) meeting the diagnostic criteria for ADHD according to the DSM-5, and (c) obtaining informed consent from both the child and their parents to participate in the study. Exclusion criteria included: (a) the presence of any psychiatric disorder other than oppositional defiant disorder, (b) the presence of a neurological or genetic disorder, (c) the presence of a chronic systemic disease (e.g., endocrinological, allergic, immunological, cardiac and hematologic disease), (d) the presence of liver or kidney failure or malignancy, (e) abnormal leukocyte and platelet values indicative of active infection (leukocytosis: >10 × 10^9^ cells/L, leukopenia: <4 × 10^9^ cells/L, thrombocytosis: >450 × 10^9^ cells/L, thrombocytopenia: <100 × 10^9^ cells/L), (f) prior use of psychotropic medication, (g) use of anti-inflammatory, antioxidant, or immunosuppressive medications within the past month, and (h) use of vitamin or dietary supplements within the last three months.

**Figure 1:**
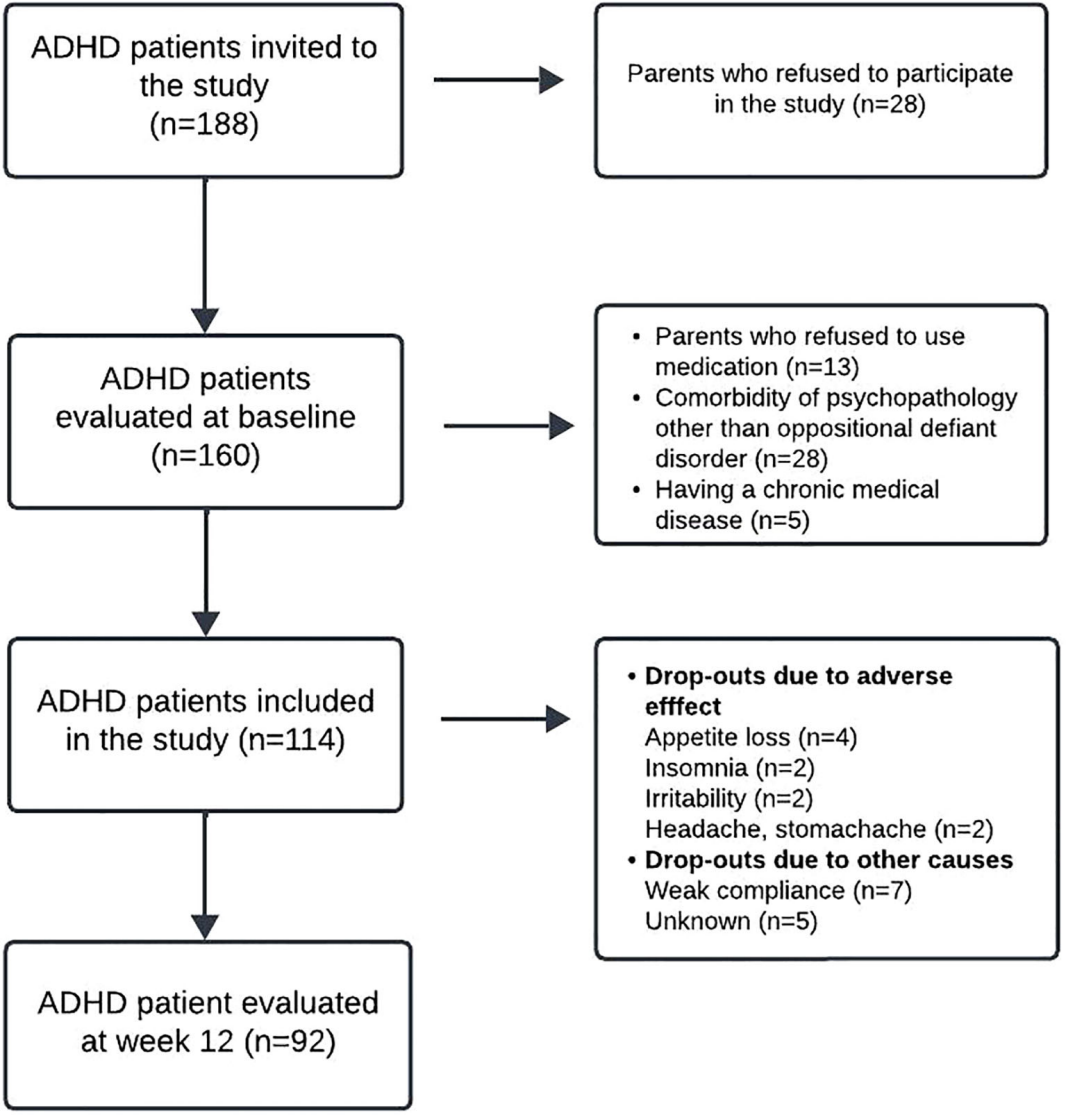
Flowchart of the study enrollment protocol.

The control group comprised psychiatrically and physically healthy children who were age—and sex-matched to the ADHD group. These children were recruited from outpatients who presented to the child psychiatry clinic for consultation and volunteered to participate in the study. To ensure the absence of any psychiatric disorder, they were screened through clinical psychiatric interviews based on DSM-5 criteria. The same exclusion criteria applied to the ADHD group were also implemented for the healthy control group. **Figure 1** illustrates a detailed overview of the study design.

The Ethics Committee of Ankara Etlik City Hospital approved the study (Protocol No: AEŞH-2024-0076). By the principles of the Declaration of Helsinki, all parents and children were informed about the study, and consent forms were signed.

### Procedure

An informative psychoeducational session was held for all participants and their parents, focusing on the effects of ADHD and its treatment on attention and behavioral control. During the session, treatment options for ADHD, including the effects and potential side effects of medications, were all explained in detail Subsequently, a demographic information form (age, gender, grade level, etc.) was completed by the ADHD and control groups. Height and weight measurements were recorded, and body mass index (BMI, kg/m²) was calculated. A semi-structured clinical interview using the Schedule for Affective Disorders and Schizophrenia for School-Age Children–Present and Lifetime Version (K-SADS-PL) was administered to all participants and their parents to assess psychiatric comorbidities. Parents of children diagnosed with ADHD completed the Conners’ Parent Rating Scale-Revised: Short Form (CPRS-R:S) and the Revised Child Anxiety and Depression Scale (RCADS).

Blood samples from all participants were collected the following morning between 8:00 AM and 9:00 AM (after 12 hours of fasting), provided there were no acute or severe illnesses.

### Treatment and assessments

#### Treatment

At recruitment, all patients were naive to any type of MPH and had received no other pharmacologic or psychological treatment. In the study, only long-acting MPH was used in the ADHD group. Parents were advised to administer the medication daily, including on weekends. The initial dose was set at 0.5 mg/kg/day, and dose adjustments were made based on treatment response and tolerability (absence of adverse symptoms).

After initiating medication, patients were assessed biweekly. In-person evaluations were conducted at weeks 0, 4, 8, and 12 in the clinic, while interim assessments were conducted via telephone. At each visit, parents were queried regarding medication adherence. Participants who missed more than three doses throughout the study were classified as non-adherent and subsequently excluded from the analyses.

Blood samples were collected from ADHD patients at baseline and week 12 to evaluate NHR, LHR, MHR, PHR, and SII levels. At these time points, clinicians assessed ADHD symptom severity and anxiety and depression symptom levels using the CPRS-R: S (27) and RCADS (28), respectively.

### Measurement tools

#### Laboratory procedures

Venous blood samples were analyzed to measure total cholesterol, HDL, low-density lipoprotein (LDL), triglycerides (TG), and CBC-derived values, including monocyte, neutrophil, platelet, and lymphocyte counts. Hemogram parameters were recorded in x10^9^/L, and HDL levels were expressed in mmol/L.

The NHR was determined by dividing the neutrophil count by the HDL level. At the same time, the MHR, LHR, and platelet-to-HDL ratio (PHR) were similarly calculated by dividing respective cell counts by HDL levels. SII, a novel indicator of immune activity and inflammatory status, was calculated using the formula: (Platelet count) × (Neutrophil count/Lymphocyte count).

### Body mass index

BMI (kg/m²) was calculated, and age-specific percentiles were determined based on the World Health Organization (WHO) growth reference curves (http://www.who.int/growthref/who2007_bmi_for_age/en/, Accessed on 01/11/2024). Using BMI percentile cut-off points specific to Turkish children, values below the 5th percentile were categorized as underweight, those between the 5th and 85th percentiles as normal weight, and values above the 85th percentile as overweight (29).

### Conners’ parent rating scale-revised: short form

The CPRS-R:S (27), is a 27-item, 4-point Likert scale designed to assess ADHD-related symptoms. It generates four independent subscale scores: cognitive problems/inattention (6 items), hyperactivity (6 items), oppositional behavior (3 items), and the ADHD index (12 items). Each item is rated from 0 (“never”) to 3 (“very often”). This study asked parents to assess their children’s behaviors using this scale. The Turkish adaptation of the scale has been validated and shown to be reliable by Kaner et al. (30). The original version of the scale exhibited high reliability and validity, with Cronbach’s alpha coefficients reported between 0.86 and 0.94.

### Revised child anxiety and depression scale

The Revised Child Anxiety and Depression Scale (RCADS), developed by Chorpita et al. (28), is a validated screening tool designed to assess anxiety disorders and depression in children and adolescents. The instrument is available in two versions: one completed by children and the other by parents, both comprising 47 items. Each item is rated on a 4-point Likert scale, ranging from 0 (“never”) to 3 (“always”). The RCADS includes six subscales that correspond to specific anxiety disorders, and it provides total scores for both anxiety and anxiety-depression by summing the relevant subscale scores. In this study, the parent version of the RCADS was utilized to evaluate participants. The Turkish adaptation of the scale was validated by Görmez et al. (31). In the original validation study, Cronbach’s alpha coefficients ranged from.78 for separation anxiety disorder to.88 for generalized anxiety disorder (28).

### Statistical analysis

Data were analyzed using SPSS^®^ 20.0 (SPSS Inc., Chicago, IL, USA). Continuous variables were presented as mean and standard deviation, while categorical variables were expressed as percentages and frequencies. The Kolmogorov-Smirnov test was used to determine whether the data followed a normal distribution. Group comparisons were conducted using the Student’s t-test for normally distributed parameters, whereas the Mann-Whitney U-test was applied to non-normally distributed parameters. Categorical variables were compared using the appropriate chi-square or Fisher’s exact test. Correlation analyses between inflammatory markers and ADHD-related psychometric scores (CPRS-R:S) were conducted. Pearson’s correlation coefficient was used for normally distributed data to evaluate linear relationships between two variables, and Spearman’s correlation coefficient was applied for non-normally distributed data. Comparisons of dependent variables at two-time points (pre- and post-treatment) were conducted using the paired-samples t-test for normally distributed variables and the Wilcoxon signed-rank test for non-normally distributed variables. For comparisons among ADHD subtypes, one-way ANOVA was performed for variables with normal distribution, and the Kruskal-Wallis test was used for variables without normal distribution. Additionally, binary logistic regression analyses were conducted to explore the relationships between NHR, LHR, MHR, PHR, and SII and the presence of ADHD. Before the regression analyses, the presence of multicollinearity among the parameters was evaluated using correlation analysis (r > 0.7). Model fit was assessed using the Hosmer-Lemeshow test, with p > 0.05 indicating a good fit. Nagelkerke’s pseudo-R² coefficient was used to evaluate the model’s predictive ability. A p-value of less than 0.05 was considered statistically significant across all analyses.

## Results

This study included 114 patients with ADHD and 52 age- and gender-matched healthy controls. The mean age of the ADHD group was 8.8 ± 1.76 years (range: 6–12 years), with 79 (69.3%) males and 35 (30.7%) females. The control group had a mean age of 9.05 ± 1.84 years (range: 6–12 years), comprising 34 (65.4%) males and 18 (34.6%) females. There were no statistically significant differences in mean age (p = 0.450) or gender distribution (p = 0.747) between the ADHD and control groups. Similarly, BMI and BMI percentiles showed no significant differences between the groups (p = 0.667 and p = 0.583, respectively). Prior to treatment, 27.2% (n = 31) of the ADHD group were categorized as having the inattentive subtype, 2.4% (n = 4) as the hyperactive-impulsive subtype, and 69.3% (n = 79) as the combined subtype.

During the pre-treatment evaluation, the ADHD group exhibited significantly higher levels of NHR, LHR, MHR, and PHR than controls (p = ≤ 0.001, p = 0.005, p = 0.003, and p = ≤ 0.001, respectively). In addition, a significant difference in the SII levels was observed between the two groups (p = 0.031). Detailed demographic and blood count parameters for both groups are provided in **Table 1**.

**Table 1:** Comparison of demographic and complete blood count parameters between ADHD patients and healthy controls.

Variables	ADHD (n=114)	Control (n=52)	χ2/Z/t	p	Effect size
Gender (male)	79 (69.3%)	34 (65.4%)	0.104	0.747	0.04
Age (years)	8.8 ±1.76	9.05±1.84	-0.756	0.450	0.06
BMI (kg/m^2^)	17.46±3.02	16.82±1.78	-0.430	0.667	0.03
BMI_Percentil	54.69±31.36	51.92±21.62	-0.548	0.583	0.04
HDL (mmol/L)	1.45±0.28	1.57±0.17	-3.521	**<0.001**	0.27
NEU (×10^9^/L)	3.59±1.10	3.00±0.76	-3.128	**0.020**	0.24
LYM (×10^9^/L)	2.86±0.56	2.83±0.6	0.268	0.789	0.05
MON (×10^9^/L)	0.55±0.2	0.49±0.13	-1.721	0.085	0.13
PLT (×10^9^/L)	331.83±61.07	330.67±47.37	0.133	0.894	0.02
NHR	2.59±0.98	1.93±0.55	-4.162	**<0.001**	0.32
LHR	2.05±0.57	1.82±0.45	-2.817	**0.005**	0.22
MHR	0.4±0.2	0.32±0.1	-2.958	**0.003**	0.23
PHR	238.07±65.37	202.11±34.11	3.356	**<0.001**	0.69
SII	437.86±202.22	368.60±149.88	-2.157	**0.031**	0.17

Pearson Chi-square (χ2), Mann-Whitney U test (Z) and student’s t test (t) were used for between-group analyses. Cohen d was used to calculate effect size. p < 0.05 was statistically significant and demonstrated in bold.

Hemogram parameters were measured in x10^9^/L, and HDL was measured in mmol/L.

ADHD, Attention Deficit Hyperactivity Disorder; BMI, body mass index; HDL, high-density lipoprotein; NEU, neutrophils; LYM, lymphocyte; MON, monocytes; PLT, platelet; NHR, neutrophil/high-density lipoprotein ratio; LHR, lymphocyte/high-density lipoprotein ratio; MHR, monocyte/high-density lipoprotein ratio; PHR, platelet/high-density lipoprotein ratio; SII, systemic inflammatory index; Values are mean and standard deviation (SD).

An analysis of blood parameters in the ADHD group, categorized by predominant presentation, revealed no significant differences in NHR, LHR, MHR, PHR, and SII levels prior to treatment (p > 0.05) (**Table 2**).

**Table 2:** Pre-treatment and post-treatment blood parameters according to ADHD predominantly appearance.

Variables	Pre-treatment						Post-treatment						
	ADHD-IA (n=31)	ADHD-HI (n =4)	ADHD-C (n=79)	F/KWH	p	Effect size	ADHD-IA (n=25)	ADHD-HI (n =4)	ADHD-C (n=63)	F/KWH	p	*Post-hoc* differences	Effect size
NHR	2.84±1.19	2.55±1.22	2.50±0.86	1.525	0.466	0.013	1.93±0.83	1.78±0.82	1.95±0.6	0.800	0.670	–	0.008
LHR	1.97±0.54	1.74±0.33	2.10±0.59	3.237	0.198	0.029	1.47±0.40	1.47±0.39	1.77±0.52	8.346	0.015	C>IA=HI	0.091
MHR	0.39±0.16	0.31±0.1	0.41±0.22	1.322	0.516	0.012	0.48±1.09	0.19±0.07	0.31±0.17	4.178	0.124	–	0.001
PHR	233.54±66.50	238.33±47.64	239.84±66.27	0.102	0.903	0.002	186.56±38.32	194.25±25.0	203.15±57.	0.932	0.398	–	0.021
SII	496.06±289.50	544.96±220.78	409.60±149.63	2.135	0.344	0.019	427.24±218.97	285.05±157.41	368.28±130.28	1.122	0.571	–	0.006

One way ANOVA (F) or Kruskal-Wallis test (KWH) were used for between-group analyses and all were supported by *post-hoc* analyses. Cohen d was used to calculate effect size. p < 0.05 was statistically significant.

Hemogram parameters were measured in x109/L, and HDL was measured in mmol/L.

NHR, neutrophil/high-density lipoprotein ratio; LHR, lymphocyte/high-density lipoprotein ratio; MHR, monocyte/high-density lipoprotein ratio; PHR, platelet/high-density lipoprotein ratio; SII, systemic inflammatory index; CPRS, Conners Parent Rating Scale; ADHD, Attention Deficit Hyperactivity Disorder; IA, inattentive, HI, hyperactive/impulsive; C, combined; Values are mean and standard deviation (SD)

Associations between inflammatory parameters and CPRS-R:S subtest scores, including the total score, were analyzed while controlling for age, gender, BMI, and total cholesterol. A significant positive correlation was found between CPRS-cognitive problems/inattention scores and PHR (p = 0.008, r = 0.253). In addition, CPRS-ADHD Index scores were significantly correlated with both the MHR (p = 0.046, r = 0.191) and PHR (p = 0.013, r = 0.237). The CPRS-Total score also significantly correlated with PHR (p = 0.025, r = 0.214). However, no significant correlations were found between the other CPRS subtest, total scores, and inflammatory parameters. The detailed results of partial correlation analyses are shown in **Table 3**.

**Table 3:** Partial correlation of pre-treatment inflammatory parameters with CPRS-R:S scores in ADHD group.

Correlation	NHR	LHR	MHR	PHR	SII
Oppositional					
*r_p_*	0.033	-0.081	-0.006	0.121	0.105
*p*	0.733	0.397	0.953	0.207	0.275
Cognitive problems/inattention					
*r_p_*	0.121	0.084	0.182	0.253	0.114
*p*	0.209	0.384	0.580	*0.008*	0.234
Hyperactivity					
*r_p_*	-0.093	-0.006	0.000	0.146	-0.027
*p*	0.333	0.948	0.999	0.127	0.781
ADHD index					
*r_p_*	0.093	0.018	0.191	0.237	0.084
*p*	0.333	0.853	*0.046*	*0.013*	0.385
Total Score					
*r_p_*	0.033	-0.008	0.105	0.214	0.076
p	0.732	0.931	0.275	*0.025*	0.432

Pearson/Spearman Correlation Analysis: r_p:_ Partial correlations were controlled for age, gender, BMI, and total cholesterol (TC, mg/dL), p < 0.05 was statistically significant and demonstrated as italic.

Hemogram parameters were measured in x109/L, and HDL was measured in mmol/L.

ADHD, Attention Deficit Hyperactivity Disorder; CPRS-R:S, Conners’ Parent Rating Scale-Revised: Short Form; NHR, Neutrophil/high-density lipoprotein (HDL) ratio; LHR, lymphocyte/HDL ratio; MHR, monocyte/HDL ratio; PHR, platelet/HDL ratio; SII, systemic inflammatory index.

Binary logistic regression analysis was conducted using the enter method on baseline (pre-treatment) data in the ADHD group. The variables included in the model were NHR, LHR, MHR, and PHR. The SII marker was excluded from the analysis due to its high correlation with NHR and LHR, indicating potential multicollinearity. Model fit was evaluated using Hosmer-Lemeshow goodness-of-fit statistics (p = 0.439). The model’s explanatory power was assessed using Cox & Snell R² and Nagelkerke R² (0.127 and 0.179, respectively). NHR was identified as an independent influencing factor for ADHD (p=0.004). **Table 4** shows logistic regression analysis results.

**Table 4:** Logistic regression analysis of the effect of NHR, LHR, MHR, and PHR on patients with ADHD.

Variables	B	S.E.	*p*	OR	OR 95% Cl
NHR	-1.012	0.351	0.004	0.363	0.183-0.722
LHR	-0.367	0.472	0.438	0.693	0.275-1.749
MHR	-0.248	1.759	0.888	0.781	0.025-24.520
PHR	0.002	0.005	0.683	1.002	0.993-1.011

Cox & Snell R² = 0.127 and Nagelkerke R^2^ = 0.179, p <0.001

Model fit was investigated by Hosmer-Lemeshow goodness-of-fit statistics (p = 0.439).

ADHD, Attention Deficit Hyperactivity Disorder; NHR, neutrophil/high-density lipoprotein (HDL) ratio; LHR, lymphocyte/HDL ratio; MHR, monocyte/HDL ratio; PHR, platelet/HDL ratio.

Of the 114 patients initially enrolled in the ADHD group, 22 discontinued treatment due to non-adherence or adverse effects. Accordingly, follow-up analyses were conducted on the remaining 92 participants who completed the 12-week study protocol. The mean initial dose of methylphenidate was 15.72 ± 5.75 mg/day, and the final dose at the end of the 12^th^ week was 23.64 ± 7.55 mg/day.

After 12 weeks of long-acting methylphenidate treatment in ADHD patients (n = 92), NHR, LHR, MHR, and PHR levels were significantly reduced compared to pre-treatment levels (p ≤ 0.001 for all). SII levels also significantly decreased following treatment (p = 0.002). The comparison of inflammatory markers, CPRS-R:S, and RCADS total anxiety and depression scores before and after treatment is provided in **Table 5**.

**Table 5:** The comparison of complete blood count parameters and CPRS and RCADS-total scores before and after treatment in patients with ADHD.

Variables	Pre-treatment (n=92)	Post-treatment (n=92)	t/Z	p	Effect size
Weight (kg)	30.81±8.73	30.68±9.46	-0.770	0.441	0.08
HDL (mmol/L)	1.45±0.28	1.62±0.23	9.009	<0.01	0.94
NEU (×10^9^/L)	3.59±1.10	3.10±0.89	-5.465	<0.01	0.57
LYM (×10^9^/L)	2.86±0.56	2.69±0.70	-3.515	<0.01	0.37
MON (×10^9^/L)	0.55±0.2	0.54±0.81	-5.765	<0.01	0.60
PLT (×10^9^/L)	331.83±61.07	318.17±51.48	-3.891	<0.01	0.41
NHR	2.59±0.98	1.94±0.67	-7.395	<0.01	0.77
LHR	2.05±0.57	1.67±0.50	-6.686	<0.01	0.70
MHR	0.4±0.2	0.35±0.58	-6.881	<0.01	0.72
PHR	236.72±64.98	198.25±51.73	8.680	<0.01	0.91
SII	437.86±202.22	380.68±161.65	-3.162	0.002	0.17
CPRS					
Oppositional	10.6±5.10	6.49±3.12	-7.277	<0.01	0.76
Cognitive problems/inattention	12.35±3.90	6.73±2.57	-8.096	<0.01	0.84
Hyperactivity	9.67±5.15	5.71±3.12	-7.595	<0.01	0.79
ADHD index	24.06±6.42	14.09±4.62	-8.200	<0.01	0.85
Total	50.68±15.55	29.74±9.93	-8.330	<0.01	0.87
RCADS_total anxiety and depression scores	32.66±19.21	22.41±12.29	-7.820	<0.01	0.81

Paired-samples t-test (t) and the Wilcoxon signed-rank test (Z) were used for examining the pre-test and post-test analyses between dependent groups. Cohen d was used to calculate effect size. p < 0.05 was statistically significant.

Hemogram parameters were measured in x109/L, and HDL was measured in mmol/L.

ADHD, Attention Deficit Hyperactivity Disorder; HDL, high-density lipoprotein; NEU, neutrophils; LYM, lymphocyte; MON, monocytes; PLT, platelet; NHR, neutrophil/high-density lipoprotein ratio; LHR, lymphocyte/high-density lipoprotein ratio; MHR, monocyte/high-density lipoprotein ratio; PHR, platelet/high-density lipoprotein ratio; SII, systemic inflammatory index; CPRS, Conners Parent Rating Scale; ADHD, Attention Deficit Hyperactivity Disorder; Values are mean and standard deviation (SD).

**Figure 2:**
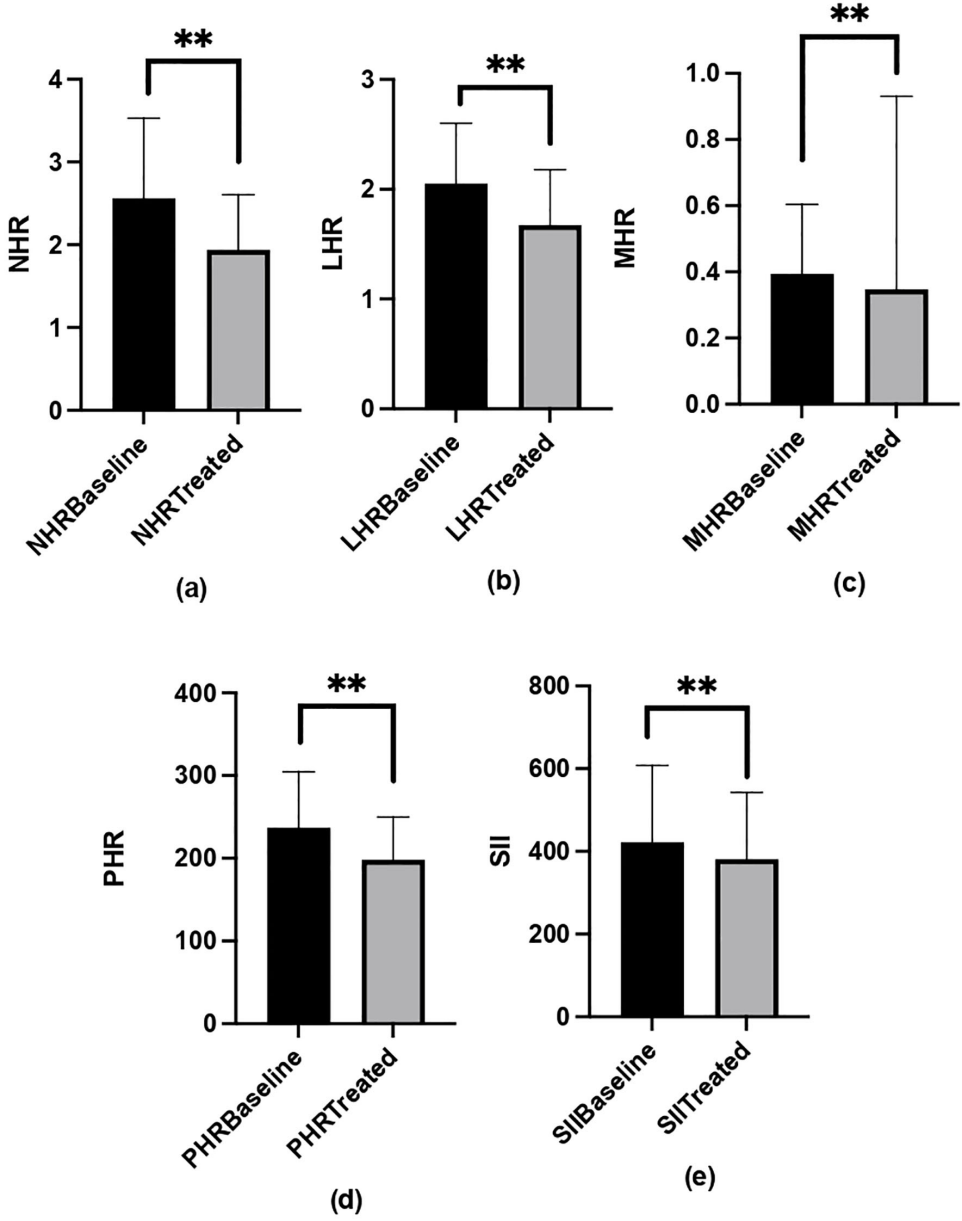
The values of NHR **(a)**, LHR **(b)**, MHR **(c)**, PHR **(d)**, and SII **(e)** in the ADHD group before and after treatment. **p<0.001.

When blood parameters in the ADHD group were analyzed based on the dominant presentation, post-treatment NHR, MHR, PHR, and SII levels did not significantly differ among subgroups (p > 0.05). However, post-treatment LHR levels were significantly higher in the combined presentation subtype compared to the inattention and hyperactivity-impulsivity subtypes (p = 0.015) (**Table 2**) (**Figure 2**).

## Discussion

This 12-week, open-label, single-center observational prospective study examined the levels of a group of novel CBC-derived inflammatory markers, including NHR, LHR, MHR, PHR, and SII, in ADHD patients compared to healthy controls, and investigated the impact of long-acting methylphenidate treatment on these markers in children aged 6–12 years. As far as we know, this is the first study to longitudinally evaluate various common hematologic parameters related to inflammation in children with ADHD. Baseline comparisons revealed significant differences in NHR, LHR, MHR, PHR and SII levels between patients with ADHD and healthy controls. After 12 weeks of long-acting methylphenidate treatment, significant reductions in NHR, LHR, MHR, PHR and SII levels were observed in patients with ADHD. Following treatment, LHR levels were found to be higher in ADHD-C compared to other subtypes. However, no significant differences were observed in other markers either before or after treatment among ADHD subtypes.

NHR, LHR, MHR, and PHR are comprehensive markers of systemic inflammation derived from routine CBC parameters, incorporating measurements such as HDL, neutrophils, lymphocytes, monocytes, and platelets. These components are closely linked to inflammation and oxidative stress (32, 33). Neutrophils, as key cells of the innate immune system, play a critical role in various physiological and pathological processes, including inflammation and autoimmunity, acting as the first line of cellular defense (34). Lymphocytes, the central components of the adaptive immune system, are pivotal in generating pathogen-specific immune responses, establishing immunological memory, and maintaining immune regulation (35). Monocytes, also essential to adaptive immunity, are involved in phagocytosis, antigen presentation, and the secretion of pro-inflammatory and pro-oxidant cytokines. By migrating to the brain and interacting with microglia, monocytes contribute to neuroinflammation (36). Platelets are critical components of both innate and adaptive immunity and function in various physiological and pathological conditions, including endothelial permeability, recruitment of neutrophils and macrophages, and as sources of cytokines and pro-inflammatory molecules (37). HDL, on the other hand, exerts antioxidant, anti-inflammatory, anti-infectious, anti-thrombotic, and immunomodulatory effects through multiple pathways, including the reduction of inflammatory responses in monocytes, platelet aggregation, lipid peroxidation, and pro-inflammatory cytokines such as TNF-α, IL-6, and IFN-γ (38). Considering the intricate interplay among neutrophils, lymphocytes, monocytes, platelets, and HDL, novel composite indices such as NHR, LHR, MHR, and PHR have gained recognition as promising biomarkers for assessing systemic inflammation levels. In recent years, there has been a growing body of research focusing on the role of systemic inflammation and immune dysregulation in the pathogenesis of ADHD (39). It has been found that inflammatory and autoimmune conditions such as atopic dermatitis, asthma, rheumatoid arthritis, type 1 diabetes and hypothyroidism may have higher odds of developing in individuals with ADHD (40). Furthermore, genetic studies have identified associations between ADHD and polymorphisms in genes linked to inflammatory pathways (41). These findings collectively suggest that inflammation may play a significant role in the etiology of ADHD and provide a robust foundation for further investigation in this field. In addition, NHR, LHR, MHR, PHR, and SII have been increasingly recognized as easy-to-measure biomarkers of systemic inflammation in various diseases (42, 43). These markers not only reflect the balance of immune responses but are also considered independent predictors in inflammatory diseases such as cancer, coronary heart disease, and COVID-19 (44, 45). These ratios, employed in other fields of biology, have recently been demonstrated in studies to be effective in identifying the role of inflammation in the etiopathogenesis of varied psychiatric conditions (14, 46).

Recent studies have shown that patients with schizophrenia, bipolar disorder, depressive disorder, and obsessive-compulsive disorder exhibit significantly higher NHR, LHR, MHR, and PHR values compared to healthy adults (9, 14, 15, 46, 47). However, no studies in the literature evaluate NHR, MHR, LHR, and PHR values in patients with ADHD. In this study, children with ADHD were found to have significantly higher NHR, MHR, LHR, and PHR values and lower HDL cholesterol levels compared to healthy controls. Furthermore, multivariate logistic regression analysis revealed that only higher NHR values were significantly associated with an ADHD diagnosis. These findings may be linked to the anti-inflammatory effects of HDL, as highlighted by Cameron et al. (2015) (48) and Nazir et al. (2020) (49). These results further support the hypothesis that inflammation may play a role in the pathogenesis of ADHD, emphasizing the importance of investigating inflammatory processes in ADHD in greater detail.

The SII has increasingly been recognized as a practical biomarker for systemic inflammation. Previous studies have demonstrated the significant role of SII in predicting the prognosis of patients with physical conditions such as tumors, neurological and cardiac diseases, and acute pancreatitis (21, 50, 51). Growing evidence supporting the role of inflammation in the etiology of psychiatric disorders has led researchers to investigate biomarkers like SII in mental health conditions. Recent studies have shown that patients with schizophrenia, bipolar disorder, depressive disorder, anxiety, and substance use disorders have significantly higher SII levels compared to healthy controls (9, 52–54). These findings suggest that SII may provide valuable insights into the pathophysiology of psychiatric disorders. In the child and adolescent population, however, there is a limited number of studies examining the relationship between SII and neurodevelopmental disorders such as ADHD and specific learning disorders (SLD). For example, in a study by Avşar et al., comparing 90 patients with SLD and 90 healthy controls, SII values were reported to be significantly higher in the patient group (23). Similarly, Öz et al. evaluated inflammatory markers in 22 newly diagnosed children with ADHD and 21 healthy controls and reported significantly higher SII levels in the ADHD group (55). Consistent with the existing literature, this study also found significantly elevated SII levels in children with ADHD compared to healthy controls. These findings suggest that inflammation may play a potential role in the pathophysiology of ADHD.

In our study, no significant relationship was found between pre-treatment NHR, LHR, MHR, PHR, and specific ADHD subtypes. Our findings align with previous research on inflammatory markers in ADHD. For instance, studies conducted on adults with ADHD have demonstrated no significant differences in IL-6, TNF-α, and morning cortisol levels between the combined and inattentive subtypes (8, 56). Similarly, a meta-analysis by Gedek et al. (2022) found no significant differences in inflammatory markers such as NLR, MLR, and PLR among ADHD subtypes (57). These findings suggest that increased inflammation may not be specific to particular ADHD subtypes but rather a general feature of ADHD as a whole. The data support the idea that inflammation may be associated with the underlying general pathophysiological mechanisms of ADHD and may be observed across all ADHD subtypes, independent of subtype differences. However, the interpretation of these findings should be cautiously approached due to the small sample sizes in certain subgroups, particularly the hyperactive-impulsive subtype (n = 4), which may have limited the statistical power of the ANOVA analysis. This limitation may reduce the reliability of the subgroup comparisons, and future studies with larger and more balanced subtype distributions are needed to validate these results.

In this study, post-treatment levels of NHR, LHR, MLR, PHR, and SII in the ADHD group were significantly lower compared to pre-treatment levels. The existing literature underscores the importance of further research to evaluate changes in these biomarkers before and after pharmacological treatment in patients with ADHD, aiming to enhance our understanding of the role of inflammation in its etiology (58). Current studies investigating the effects of psychostimulants on oxidative stress and inflammation yield inconsistent findings, largely derived from animal models or measurements conducted on different samples and brain regions. The effects of methylphenidate have been shown to vary depending on age, duration of treatment, and brain region. For instance, acute MPH treatment in young rats reduced lipid peroxidation (LPO), whereas chronic treatment increased oxidative damage; in adult rats, however, chronic treatment reduced oxidative stress (59). Regional differences in the brain were also observed, with oxidative stress decreasing in some areas (e.g., the striatum and cerebellum) but worsening in the prefrontal cortex (60). In ADHD animal models, the therapeutic use of MPH increased antioxidant capacity and reduced oxidative damage. In contrast, non-therapeutic use of MPH in healthy rats increased oxidative and nitrosative stress and produced pro-inflammatory effects. In addition, MPH treatment improved the pro-inflammatory profile in ADHD models while inducing a pro-inflammatory state under control conditions (61). These findings suggest that the effects of MPH depend on the context and mode of use. Limited studies have shown that MPH treatment not only improves oxidative and inflammatory conditions but also enhances hypothalamic-pituitary-adrenal (HPA) axis function in children with ADHD (7, 58, 62). The findings of this study align with clinical research, further supporting the notion that MPH reduces inflammation in ADHD. Future studies should incorporate extended treatment durations, explore the effects of other forms of MPH, and examine the impact of treating comorbid conditions to provide deeper insights into this association.

This study should be evaluated in light of its strengths and limitations. Among its strengths, the prospective design employed for children with ADHD, the use of standardized and validated assessment tools (CPRS-R:S, RCADS), and the evaluation of biological parameters (lipid profile, inflammatory markers) are notable. In addition, including a control group allowed for comparative analyses, enhancing the study’s methodological rigor. However, several limitations should also be considered. First, the study was conducted in a single center and employed an open-label design, which may limit the generalizability and introduce potential observer bias. Second, the narrow age range of the sample and the exclusion of psychiatric comorbidities (e.g., disorders other than oppositional defiant disorder) and chronic medical conditions ensured a focus on a homogeneous ADHD group but simultaneously excluded the common real-world occurrence of ADHD with comorbid medical and psychiatric conditions. This may limit the applicability of the findings to routine clinical practice. Furthermore, the reliance solely on parent-reported measures, without incorporating teacher or self-reported assessments, increases the risk of single-source bias. The 12-week duration of this study may have been insufficient to assess the long-term effects of extended-release MPH on inflammation comprehensively. Additionally, this study did not include several promising inflammatory biomarkers, and there was an absence of measurement for potential confounding variables such as dietary habits and physical activity, which may have influenced the outcomes.

## Conclusions

This study provides significant insights into the relationship between ADHD and systemic inflammation. It demonstrated that a 12-week treatment with long-acting MPH significantly reduced inflammatory markers such as NHR, LHR, MHR, PHR, and SII. These findings suggest that MPH not only alleviates core symptoms of ADHD but also modulates inflammatory processes, highlighting the potential role of inflammation in the pathophysiology of ADHD. Furthermore, the study emphasizes that hematologic ratios derived from routine blood tests can serve as accessible and cost-effective biomarkers for assessing systemic inflammation in ADHD. However, these findings should be considered preliminary and insufficient to support clinical application without further validation.

These findings hold important implications for both research and clinical practice. Associating ADHD treatment with changes in inflammatory markers contributes to the growing evidence supporting the role of immune dysregulation in ADHD. The observed anti-inflammatory effects of MPH underline the necessity of exploring its broader biological impacts and its potential long-term effects on ADHD outcomes.

Future studies should address the limitations of this research by focusing on the long-term effects of MPH on inflammation. In addition, incorporating advanced biomarkers such as cytokines, oxidative stress indicators, and neuroinflammatory markers could provide a more comprehensive understanding of the underlying mechanisms. Research examining the relationship between systemic inflammation and ADHD subtypes, as well as the potential impact of comorbid conditions, will further enhance our understanding of these complex interactions.

## Data Availability

The raw data supporting the conclusions of this article will be made available by the authors, without undue reservation.

## References

[1] Polanczyk G De Lima MS Horta BL Biederman J Rohde LA. The worldwide prevalence of ADHD: a systematic review and metaregression analysis. Am J Psychiatry. (2007) 164:942–8. doi: 10.1176/ajp.2007.164.6.942, PMID: 17541055

[2] Faraone SV Perlis RH Doyle AE Smoller JW Goralnick JJ Holmgren MA. Molecular genetics of attention-deficit/hyperactivity disorder. Biol Psychiatry. (2005) 57:1313–23. doi: 10.1016/j.biopsych.2004.11.024, PMID: 15950004

[3] Garre-Morata L de Haro T Villén RG Fernández-López ML Escames G Molina-Carballo A. Changes in cortisol and in oxidative/nitrosative stress indicators after ADHD treatment. Antioxidants. (2024) 13:92. doi: 10.3390/antiox13010092, PMID: 38247516 PMC10812591

[4] Corona JC. Role of oxidative stress and neuroinflammation in attention-deficit/hyperactivity disorder. Antioxidants. (2020) 9:1039. doi: 10.3390/antiox9111039, PMID: 33114154 PMC7690797

[5] Kozłowska A Wojtacha P Równiak M Kolenkiewicz M Huang ACW. ADHD pathogenesis in the immune, endocrine and nervous systems of juvenile and maturating SHR and WKY rats. Psychopharmacol (Berl). (2019) 236:2937–58. doi: 10.1007/s00213-019-5180-0, PMID: 30737597 PMC6820808

[6] Misiak B Wojta-Kempa M Samochowiec J Schiweck C Aichholzer M Reif A. Peripheral blood inflammatory markers in patients with attention deficit/hyperactivity disorder (ADHD): A systematic review and meta-analysis. Prog Neuropsychopharmacol Biol Psychiatry. (2022) 118:110581. doi: 10.1016/j.pnpbp.2022.110581, PMID: 35660454

[7] Oades RD Dauvermann MR Schimmelmann BG Schwarz MJ Myint AM. Attention-deficit hyperactivity disorder (ADHD) and glial integrity: S100B, cytokines and kynurenine metabolism-effects of medication. Behav Brain Functions. (2010) 6:1–14. doi: 10.1186/1744-9081-6-29, PMID: 20509936 PMC2889842

[8] Corominas-Roso M Armario A Palomar G Corrales M Carrasco J Richarte V. IL-6 and TNF-α in unmedicated adults with ADHD: Relationship to cortisol awakening response. Psychoneuroendocrinology. (2017) 79:67–73. doi: 10.1016/j.psyneuen.2017.02.017, PMID: 28262601

[9] Wei Y Wang T Li G Feng J Deng L Xu H. Investigation of systemic immune-inflammation index, neutrophil/high-density lipoprotein ratio, lymphocyte/high-density lipoprotein ratio, and monocyte/high-density lipoprotein ratio as indicators of inflammation in patients with schizophrenia and bipolar disorder. Front Psychiatry. (2022) 13:941728. doi: 10.3389/fpsyt.2022.941728, PMID: 35958647 PMC9360542

[10] Goossens J Morrens M Coppens V. The potential use of peripheral blood mononuclear cells as biomarkers for treatment response and outcome prediction in psychiatry: a systematic review. Mol Diagn Ther. (2021) 25:283–99. doi: 10.1007/s40291-021-00516-8, PMID: 33978935

[11] Barter PJ Nicholls S Rye KA Anantharamaiah GM Navab M Fogelman AM. Antiinflammatory properties of HDL. Circ Res. (2004) 95:764–72. doi: 10.1161/01.RES.0000146094.59640.13, PMID: 15486323

[12] Xu H Li R Wang L Wang T Luo Y Wei Y. Non-enzymatic antioxidants, macro-minerals and monocyte/high-density lipoprotein cholesterol ratio among patients with bipolar disorder. J Affect Disord. (2023) 322:76–83. doi: 10.1016/j.jad.2022.11.017, PMID: 36372130

[13] Jialal I Jialal G Adams-Huet B. The platelet to high density lipoprotein-cholesterol ratio is a valid biomarker of nascent metabolic syndrome. Diabetes Metab Res Rev. (2021) 37:e3403. doi: 10.1002/dmrr.3403, PMID: 32886844

[14] Villegas García L Patró E Barbero JD Esteve-Valverde E Palao DJ Soria V. Lymphocyte-derived and lipoprotein-derived inflammatory ratios as biomarkers in bipolar disorder type I: Characteristics, predictive values, and influence of current psychopharmacological treatments. Psychoneuroendocrinology. (2025) 171:107209. doi: 10.1016/j.psyneuen.2024.107209, PMID: 39442230

[15] Korkmaz ŞA Kızgın S. Neutrophil/high-density lipoprotein cholesterol (HDL), monocyte/HDL and platelet/HDL ratios are increased in acute mania as markers of inflammation, even after controlling for confounding factors. Curr Med Res Opin. (2023) 39:1383–90. doi: 10.1080/03007995.2023.2260302, PMID: 37725087

[16] Wei Y Gao H Luo Y Feng J Li G Wang T. Systemic inflammation and oxidative stress markers in patients with unipolar and bipolar depression: a large-scale study. J Affect Disord. (2024) 346:154–66. doi: 10.1016/j.jad.2023.10.156, PMID: 37924985

[17] Cheng N Ma H Zhang K Zhang C Geng D. The predictive value of monocyte/high-density lipoprotein ratio (MHR) and positive symptom scores for aggression in patients with schizophrenia. Medicina. (2023) 59:503. doi: 10.3390/medicina59030503, PMID: 36984504 PMC10055014

[18] Qing G Bao C Yang Y Wei B. Association between neutrophil to high-density lipoprotein cholesterol ratio (NHR) and depression symptoms among the United States adults: a cross-sectional study. Lipids Health Dis. (2024) 23:215. doi: 10.1186/s12944-024-02204-y, PMID: 39003458 PMC11245866

[19] Zhang H Xu Y Xu Y. The value of the platelet/high-density lipoprotein cholesterol ratio in predicting depression and its cardiovascular disease mortality: a population-based observational study. Front Endocrinol. (2024) 15:1402336. doi: 10.3389/fendo.2024.1402336, PMID: 39149124 PMC11325088

[20] Hu B Yang XR Xu Y Sun YF Sun C Guo W. Systemic immune-inflammation index predicts prognosis of patients after curative resection for hepatocellular carcinoma. Clin Cancer Res. (2014) 20:6212–22. doi: 10.1158/1078-0432.CCR-14-0442, PMID: 25271081

[21] Liu X Guan G Cui X Liu Y Liu Y Luo F. Systemic immune-inflammation index (SII) can be an early indicator for predicting the severity of acute pancreatitis: a retrospective study. Int J Gen Med. (2021) 14:9483–9. doi: 10.2147/IJGM.S343110, PMID: 34949937 PMC8689009

[22] Ceyhun HA Gürbüzer N. New hematological parameters as inflammatory biomarkers: Systemic immune inflammation index, platerethritis, and platelet distribution width in patients with adult attention deficit hyperactivity disorder. Adv Neurodev Disord. (2022) 6:211–23. doi: 10.1007/s41252-022-00258-6, PMID: 35573104 PMC9091147

[23] Avşar PA Kara T Kocaman O Akkuş M. Evaluation of primary markers of inflammation and the systemic inflammation index in specific learning disabilities. biomark Med. (2024) 18:907–16. doi: 10.1080/17520363.2024.2404387, PMID: 39360657 PMC11509046

[24] Pliszka S Issues AWG on Q. Practice parameter for the assessment and treatment of children and adolescents with attention-deficit/hyperactivity disorder. J Am Acad Child Adolesc Psychiatry. (2007) 46:894–921. doi: 10.1097/chi.0b013e318054e724, PMID: 17581453

[25] da Silva BS Leffa DT Beys-da-Silva WO Torres ILS Rovaris DL Victor MM. Integrative proteomics and pharmacogenomics analysis of methylphenidate treatment response. Transl Psychiatry. (2019) 9:308. doi: 10.1038/s41398-019-0649-5, PMID: 31740662 PMC6861257

[26] Foschiera LN Schmitz F Wyse ATS. Evidence of methylphenidate effect on mitochondria, redox homeostasis, and inflammatory aspects: Insights from animal studies. Prog Neuropsychopharmacol Biol Psychiatry. (2022) 116:110518. doi: 10.1016/j.pnpbp.2022.110518, PMID: 35092763

[27] Conners CK Sitarenios G Parker JDA Epstein JN. The revised Conners’ Parent Rating Scale (CPRS-R): factor structure, reliability, and criterion validity. J Abnorm Child Psychol. (1998) 26:257–68. doi: 10.1023/A:1022602400621, PMID: 9700518

[28] Chorpita BF Yim L Moffitt C Umemoto LA Francis SE. Assessment of symptoms of DSM-IV anxiety and depression in children: A revised child anxiety and depression scale. Behav Res Ther. (2000) 38:835–55. doi: 10.1016/S0005-7967(99)00130-8, PMID: 10937431

[29] Neyzi O Furman A Bundak R Gunoz H Darendeliler F Bas F. Growth references for Turkish children aged 6 to 18 years. Acta Paediatr. (2006) 95:1635–41. doi: 10.1080/08035250600652013, PMID: 17129974

[30] Kaner S Buyukozturk S Iseri E. Conners parent rating scale-revised short: Turkish standardization study. Arch Neuropsychiatry. (2013) 50:100–10. doi: 10.4274/npa.y6219

[31] Gormez V Kılınçaslan A Orengul AC Ebesutani C Kaya I Ceri V. Psychometric properties of the Turkish version of the Revised Child Anxiety and Depression Scale–Child Version in a clinical sample. Psychiatry Clin Psychopharmacology. (2017) 27:84–92. doi: 10.1080/24750573.2017.1297494 28251450

[32] Tang H Xiang Z Li L Shao X Zhou Q You X. Potential role of anti-inflammatory HDL subclasses in metabolic unhealth/obesity. Artif Cells Nanomed Biotechnol. (2021) 49:564–74. doi: 10.1080/21691401.2021.1961798, PMID: 34402692

[33] Cheng Y Wang Y Wang X Jiang Z Zhu L Fang S. Neutrophil-to-lymphocyte ratio, platelet-to-lymphocyte ratio, and monocyte-to-lymphocyte ratio in depression: an updated systematic review and meta-analysis. Front Psychiatry. (2022) 13:893097. doi: 10.3389/fpsyt.2022.893097, PMID: 35782448 PMC9240476

[34] Liew PX Kubes P. The neutrophil’s role during health and disease. Physiol Rev. (2019) 99:1223–48. doi: 10.1152/physrev.00012.2018, PMID: 30758246

[35] Bonilla FA Oettgen HC. Adaptive immunity. J Allergy Clin Immunol. (2010) 125:S33–40. doi: 10.1016/j.jaci.2009.09.017, PMID: 20061006

[36] Wohleb ES Fenn AM Pacenta AM Powell ND Sheridan JF Godbout JP. Peripheral innate immune challenge exaggerated microglia activation, increased the number of inflammatory CNS macrophages, and prolonged social withdrawal in socially defeated mice. Psychoneuroendocrinology. (2012) 37:1491–505. doi: 10.1016/j.psyneuen.2012.02.003, PMID: 22386198 PMC3368999

[37] Pogorzelska K Krętowska A Krawczuk-Rybak M Sawicka-Żukowska M. Characteristics of platelet indices and their prognostic significance in selected medical condition–a systematic review. Adv Med Sci. (2020) 65:310–5. doi: 10.1016/j.advms.2020.05.002, PMID: 32505856

[38] Barker G Leeuwenburgh C Brusko T Moldawer L Reddy ST Guirgis FW. Lipid and lipoprotein dysregulation in sepsis: Clinical and mechanistic insights into chronic critical illness. J Clin Med. (2021) 10:1693. doi: 10.3390/jcm10081693, PMID: 33920038 PMC8071007

[39] Saccaro LF Schilliger Z Perroud N Piguet C. Inflammation, anxiety, and stress in attention-deficit/hyperactivity disorder. Biomedicines. (2021) 9:1313. doi: 10.3390/biomedicines9101313, PMID: 34680430 PMC8533349

[40] Lin YT Chen YC Gau SSF Yeh TH Fan HY Hwang YY. Associations between allergic diseases and attention deficit hyperactivity/oppositional defiant disorders in children. Pediatr Res. (2016) 80:480–5. doi: 10.1038/pr.2016.111, PMID: 27356086

[41] Dunn GA Nigg JT Sullivan EL. Neuroinflammation as a risk factor for attention deficit hyperactivity disorder. Pharmacol Biochem Behav. (2019) 182:22–34. doi: 10.1016/j.pbb.2019.05.005, PMID: 31103523 PMC6855401

[42] Wei L Xie H Yan P. Prognostic value of the systemic inflammation response index in human Malignancy: a meta-analysis. Medicine. (2020) 99:e23486. doi: 10.1097/MD.0000000000023486, PMID: 33327280 PMC7738007

[43] Liu Z Fan Q Wu S Wan Y Lei Y. Compared with the monocyte to high-density lipoprotein ratio (MHR) and the neutrophil to lymphocyte ratio (NLR), the neutrophil to high-density lipoprotein ratio (NHR) is more valuable for assessing the inflammatory process in Parkinson’s disease. Lipids Health Dis. (2021) 20:1–12. doi: 10.1186/s12944-021-01462-4, PMID: 33874966 PMC8054377

[44] Liu Y Ye T Chen L Jin T Sheng Y Wu G. Systemic immune-inflammation index predicts the severity of coronary stenosis in patients with coronary heart disease. Coron Artery Dis. (2021) 32:715–20. doi: 10.1097/MCA.0000000000001037, PMID: 33826540

[45] Salman E Celikbilek N Aydoğan S Özdem B Gökay S Kirca F. Investigation of the relationship of systemic immune-inflammation index, C-Reactive protein and interleukin-6 with viral dynamics in patients with COVID-19. Mikrobiyol Bul. (2021) 55:539–52. doi: 10.5578/mb.20219706, PMID: 34666654

[46] Chen J Huang Y Li X. The association between lymphocyte to high-density lipoprotein ratio and depression: Data from NHANES 2015–2018. Brain Behav. (2024) 14:e3467. doi: 10.1002/brb3.3467, PMID: 38468463 PMC10928332

[47] Abus S Kapici Y Ayhan S Guc B Tekin A. Comparative study of frontal QRS-T angle and inflammatory parameters in obsessive compulsive disorder patients and healthy control group. Medicine. (2022) 11:1473–7. doi: 10.5455/medscience.2022.08.189

[48] Cameron SJ Morrell CN Bao C Swaim AF Rodriguez A Lowenstein CJ. A novel anti-inflammatory effect for high density lipoprotein. PloS One. (2015) 10:e0144372. doi: 10.1371/journal.pone.0144372, PMID: 26680360 PMC4683005

[49] Nazir S Jankowski V Bender G Zewinger S Rye KA van der Vorst EPC. Interaction between high-density lipoproteins and inflammation: Function matters more than concentration! Adv Drug Delivery Rev. (2020) 159:94–119. doi: 10.1016/j.addr.2020.10.006, PMID: 33080259

[50] Bittoni A Pecci F Mentrasti G Crocetti S Lupi A Lanese A. Systemic immune-inflammation index: a prognostic tiebreaker among all in advanced pancreatic cancer. Ann Transl Med. (2021) 9:251. doi: 10.21037/atm-20-3499, PMID: 33708878 PMC7940927

[51] Zhou YX Li WC Xia SH Xiang T Tang C Luo JL. Predictive value of the systemic immune inflammation index for adverse outcomes in patients with acute ischemic stroke. Front Neurol. (2022) 13:836595. doi: 10.3389/fneur.2022.836595, PMID: 35370926 PMC8971364

[52] Cui S Li J Liu Y Yao G Wu Y Liu Z. Correlation of systemic immune-inflammation index and moderate/major depression in patients with depressive disorders: a large sample cross-sectional study. Front Psychiatry. (2023) 14:1159889. doi: 10.3389/fpsyt.2023.1159889, PMID: 37275977 PMC10232846

[53] Canlı D. Evaluation of systemic immune-inflammation index, systemic inflammatory response index and hematologic inflammatory parameters in generalized anxiety disorder: a controlled study. Anatolian Curr Med J. (2024) 6:161–7. doi: 10.38053/acmj.1427475

[54] Sehlikoğlu Ş Yıldız S Kılıçaslan AK Kurt O Göçüm E Almiş BH. Evaluation of complete blood cell count parameters and their role in inflammation in patients with methamphetamine and synthetic cannabis use disorder. Psychiatry Clin Psychopharmacology. (2024) 34:134. doi: 10.5152/pcp.2024.23803, PMID: 39165890 PMC11332474

[55] Öz E Parlak ME Kapıcı Y Balatacı U Küçükkelepçe O Kurt F. Pre-and post-treatment evaluation of routine blood analysis in patients with attention deficit hyperactivity disorder and comparison with the healthy control group. Sci Rep. (2023) 13:16233. doi: 10.1038/s41598-023-43553-5, PMID: 37758832 PMC10533532

[56] Ramos-Quiroga JA Corominas-Roso M Palomar G Ferrer R Valero S Corrales M. Cortisol awakening response in adults with attention deficit hyperactivity disorder: Subtype differences and association with the emotional lability. Eur Neuropsychopharmacol. (2016) 26:1140–9. doi: 10.1016/j.euroneuro.2016.03.014, PMID: 27084305

[57] Gędek A Modrzejewski S Gędek M Antosik AZ Mierzejewski P Dominiak M. Neutrophil to lymphocyte ratio, platelet to lymphocyte ratio, and monocyte to lymphocyte ratio in ADHD: a systematic review and meta-analysis. Front Psychiatry. (2023) 14:1258868. doi: 10.3389/fpsyt.2023.1258868, PMID: 38034918 PMC10682201

[58] Verlaet AAJ Breynaert A Ceulemans B De Bruyne T Fransen E Pieters L. Oxidative stress and immune aberrancies in attention-deficit/hyperactivity disorder (ADHD): A case–control comparison. Eur Child Adolesc Psychiatry. (2019) 28:719–29. doi: 10.1007/s00787-018-1239-4, PMID: 30350094

[59] Martins MR Reinke A Petronilho FC Gomes KM Dal-Pizzol F Quevedo J. Methylphenidate treatment induces oxidative stress in young rat brain. Brain Res. (2006) 1078:189–97. doi: 10.1016/j.brainres.2006.01.004, PMID: 16494852

[60] Schmitz F Scherer EBS MaChado FR da Cunha AA Tagliari B Netto CA. Methylphenidate induces lipid and protein damage in prefrontal cortex, but not in cerebellum, striatum and hippocampus of juvenile rats. Metab Brain Dis. (2012) 27:605–12. doi: 10.1007/s11011-012-9335-5, PMID: 22968482

[61] Sanches ES Boia R Leitão RA Madeira MH Fontes-Ribeiro CA Ambrósio AF. Attention-deficit/hyperactivity disorder animal model presents retinal alterations and methylphenidate has a differential effect in ADHD versus control conditions. Antioxidants. (2023) 12:937. doi: 10.3390/antiox12040937, PMID: 37107312 PMC10135983

[62] Guney E Cetin FH Alisik M Tunca H Torun YT Iseri E. Attention deficit hyperactivity disorder and oxidative stress: a short term follow up study. Psychiatry Res. (2015) 229:310–7. doi: 10.1016/j.psychres.2015.07.003, PMID: 26188640

